# Energetic Electron-Assisted Synthesis of Tailored Magnetite (Fe_3_O_4_) and Maghemite (γ−Fe_2_O_3_) Nanoparticles: Structure and Magnetic Properties

**DOI:** 10.3390/nano13050786

**Published:** 2023-02-21

**Authors:** Johannes Dietrich, Alexius Enke, Nils Wilharm, Robert Konieczny, Andriy Lotnyk, André Anders, Stefan G. Mayr

**Affiliations:** 1 Leibniz Institute of Surface Engineering (IOM), Permoserstraße 15, 04318 Leipzig, Germany; 2 Division of Surface Physics, Faculty of Physics and Earth Science, Leipzig University, Linnéstraße 5, 04103 Leipzig, Germany; 3 Division of Applied Physics, Faculty of Physics and Earth Science, Leipzig University, Linnéstraße 5, 04103 Leipzig, Germany

**Keywords:** nanoparticles, electron beam irradiation, magnetic properties, magnetite, maghemite, microemulsion

## Abstract

Iron oxide nanoparticles with a mean size of approximately 5 nm were synthesized by irradiating micro-emulsions containing iron salts with energetic electrons. The properties of the nanoparticles were investigated using scanning electron microscopy, high-resolution transmission electron microscopy, selective area diffraction and vibrating sample magnetometry. It was found that formation of superparamagnetic nanoparticles begins at a dose of 50 kGy, though these particles show low crystallinity, and a higher portion is amorphous. With increasing doses, an increasing crystallinity and yield could be observed, which is reflected in an increasing saturation magnetization. The blocking temperature and effective anisotropy constant were determined via zero-field cooling and field cooling measurements. The particles tend to form clusters with a size of 34 nm to 73 nm. Magnetite/maghemite nanoparticles could be identified via selective area electron diffraction patterns. Additionally, goethite nanowires could be observed.

## 1. Introduction

In recent decades, synthesis of magnetic nanoparticles has been intensively developed, not only because of fundamental scientific interest, but also for their broad range of biomedical applications such as drug delivery [1], contrast agents for resonance imaging [2] and hyperthermia treatment [3]. Besides that, ferrogels, i.e., magnetically controllable gel–magnetic nanoparticle composites, are a highly promising upcoming materials class within this field [4,5,6]. All the applications have different requirements regarding the thermal, chemical, magnetic and mechanical properties of the particles, which, as with most nanomaterials, are affected by the size, shape and crystalline structure of the particle [7,8].

Due to excellent biocompatiblity and medical approval for selected applications, iron oxides, such as magnetite (Fe_3_O_4_) and maghemite (γ−Fe_2_O_3_), constitute highly attractive compounds within this scope [9,10]. Both are also characterized by attractive magnetic properties, particularly a large magnetic moment [9,11], which leads to their extraordinary ability to be controlled in a targeted manner through the use of an external magnetic field [12]. Bulk magnetite and maghemite are ferrimagnetic. They have spatially alternating magnetic moments, but the magnitude of the moments are unequal and magnetization remains. If nanoparticles consisting of these iron oxides are below a certain size, they show superparamagnetic behavior and, due to thermal relaxation, no magnetization remains after the magnetic field has been switched off [13]. For nanoparticles based on iron oxides, the critical diameter is around 10 nm to 20 nm at room temperature [9,14]. A standard method to synthesize iron oxide particles is based on alkaline precipitation from the solution of mixed Fe^2+^ and Fe^3+^ salts. This procedure provides a high yield, but also a limited control over the particle sizes and a wide size distribution [10]. As mentioned before, control of the monodisperse size is key, as the properties of the nanocrystals strongly depend on the dimension of the nanoparticles. Gamma irradiation can be used to overcome this problem and has been widely used in the past decade in order to obtain an additional parameter (dose) to control the nanoparticle size and related properties [15,16,17]. The mechanisms induced by energetic electron and gamma irradiation have some similarities; in both cases secondary electrons are created, a reducing environment is generated and reactions with solvated metal ions are initiated, such that metal ions are reduced [18,19]. The main difference is the dose rate. While gamma irradiation can take several hours, electron irradiation takes only a few minutes to obtain the same dose, which makes it an efficient and suitable tool to obtain magnetic nanoparticles. Of course, energy consumption must be taken into account, which can be higher for electron beam irradiation, but upscaling on an industrial scale would mitigate this drawback. Another advantage of these radiolytic methods is the initiation of homogeneous polymerization inside gels, which is used to modify the mechanical properties and sterilize final products [20,21,22,23]. A common procedure to synthesize ferrogels is to add already prepared magnetic nanoparticles to a gel precursor and irradiate this solution with electron or gamma irradiation to induce crosslinking of the polymer [24,25]. Simplifying this two-step process to only one process by synthesizing nanoparticles and gel at the same time has been barely studied [26] and is a key motivation for this work. A one step process would be highly attractive for upscaling and industry scale production. Additionally, a one step process intrinsically solves the problem of obtaining a homogeneous nanoparticle dispersion. The objectives of the present work are to investigate the influence of electron irradiation on the formation of nanosized magnetite/maghemite particles and their corresponding morphology, magnetic properties and crystal structure as a base for the future one-step synthesis of ferrogels.

## 2. Materials and Methods

### 2.1. Preparation of the Samples

Iron(III) chloride hexahydrate (FeCl_3_·6H_2_O), iron(II) sulfate heptahydrate (FeSO_4_·7H_2_O), Triton X-100, cyclohexane, 1-pentanol and tetramethylammonium hydroxide solution (TMAH) were supplied by Sigma-Aldrich (St. Louis, MO, USA). The sample preparation was based on the micro-emulsion method described by Yang et al. [27] and Gotić et al. [15]. The first microemulsion contained 0.5 mL aqueous solution of the iron salts (0.075 mol/L FeCl_3_, 0.05 mol/L FeSO_4_), 14 mL cyclohexane, 1.5 mL Triton X-100 and 0.5 mL 1-pentanol. The second microemulsion contained 0.5 mL aqueous solution of TMAH (1 mol/L) and again 14 mL cyclohexane, 1.5 mL Triton X-100 and 0.5 mL 1-pentanol. From each microemulsion, 1 mL was mixed creating the final 2 mL sample with a pH value between 12 and 13. This sample was bubbled for several minutes with nitrogen in order to remove oxygen.

### 2.2. Irradiation

The samples were irradiated with high energy electrons (10 MeV) by a linear accelerator from Mevex (MB10-30MP, Stittsville, ON, Canada). The accelerator had a pulse repetition rate of 460 Hz, a pulse length of 8 µs and a peak beam power of 10 kW. A total dose of 250 kGy was carried out in steps of 50 kGy. After the irradiation, 200 µL of acetone was added to each sample to promote coagulation.

### 2.3. Sample Characterization

Scanning electron microscopy (SEM) and vibrating sample magnetometer (VSM) measurements were directly performed on the irradiated samples dissolved in cyclohexane. No additional cleaning step was performed between irradiation and measurements. For SEM imaging, which was conducted by an Ultra 55 (Carl Zeiss, Oberkochen, Germany), the irradiated samples were dispersed in an ultrasound bath to obtain a homogeneous distribution, subsequently pipetted on a Si Wafer and dried in an oven at around 100 °C for up to four hours. For a comparison, non-irradiated solutions were also prepared. An amount of 15 µL of the solution was pipetted into specific sample holders for VSM measurements, which were performed with a Physical Property Measurement System (PPMS) from Quantum Design (San Diego, CA, USA). The oscillating frequency of the measurement was 40 Hz with an averaging time of 1 s. Dried powder samples were used for low temperature measurements such as zero-field cooling (ZFC) and field cooling (FC). The cooling routines were carried out at a rate of 2 K/min. Residues of the solvent or the reactants formed a layer around the nanoparticles, which limited the electron transmission through the substrate. Therefore, several cleaning steps of the precipitate by ethanol and then acetone were employed prior to transmission electron microscopy (TEM). Between each washing step, the precipitate was separated from the ethanol or acetone solvent using centrifugation. The powder was dispersed in ethanol using an ultrasound bath, then a drop of the dispersion was released on a copper grid and dried for several hours at room temperature. TEM investigations were performed using a Titan${}^{3}$ G2 60–300 (FEI Company, Hillsboro, OR, USA).

### 2.4. Framework for Evaluating Magnetization Data

Assuming superparamagnetism, magnetization curves, M(H), of superparamagnetic nanoparticles can be approximated by the Langevin Equation [28]:

(1) $$\begin{array}{c} L\left(x\right)=\coth\left(x\right)-\frac{1}{x},\;\mathrm{with}\;x=\frac{\mu H}{k_{B}T} \end{array}$$

(2) $$\begin{array}{c} \;\;\;\;\;\frac{M(H)}{M_{S}}=\coth\left(\frac{\mu H}{k_{B}T}\right)-\frac{k_{B}T}{\mu H} \end{array}$$

where $M_{S}$ is the saturation magnetization of the sample, *H* is the applied magnetic field, $k_{B}$ is the Boltzmann constant and *T* is the temperature. The magnetic moment, μ, of the particle is linked to its volume, *V*, by:

(3) $$\begin{array}{c} \mu =V\cdot M_{\mathrm{SB}} \end{array}$$

where $M_{\mathrm{SB}}$ is the saturation magnetization per volume of the corresponding bulk material. Assuming a spherical shape of the nanoparticles, one can write Equation (2) in terms of the particle diameter, *D*:

(4) $$\begin{array}{c} \frac{M(H)}{M_{S}}=\coth\left(\frac{\pi M_{\mathrm{SB}}D^{3}H}{6k_{B}T}\right)-\frac{6k_{B}T}{\pi M_{\mathrm{SB}}D^{3}H} \end{array}$$

Knowing the saturation magnetization of the bulk material, $M_{SB}$, one can obtain an approximation of the particle diameter, *D*, from the measured M(H) curves. This approximation does not take into account any particle size distribution inside the sample or the volume dependence of $M_{SB}$. Chen et al. [29] and El-Hilo et al. [30] have presented more advanced models to determine particle sizes from magnetization curves by integrating over volume-weighted and number-weighted size distributions, which will not be discussed here.

Due to the magnetic anisotropy of the nanoparticles, the magnetic moment has two possible orientations which are separated by an energy barrier KV, where *K* is the anisotropy constant (or anisotropy energy density) and *V* is the volume of the particle. Thermal fluctuations can cause a flip of the magnetization to the other orientation. The time between these two flips is called the Néel relaxation time, $\tau_{N}$ [31]:

(5) $$\begin{array}{c} \tau_{N}=\tau_{0}\exp\left(\frac{KV}{k_{B}T}\right) \end{array}$$

where $\tau_{0}$ is the intrinsic inversion time of the material and is usually in the range of $10^{-9}$ s [31,32]. The magnetic state of the nanoparticle is blocked when the measurement time, $\tau_{m}$, is shorter than $\tau_{N}$. By setting $\tau_{m}=\tau_{N}$, Equation (5) can be rewritten and one obtains the so called blocking temperature, $T_{B}$ [31]:

(6) $$\begin{array}{c} T_{B}=\frac{KV}{k_{B}\ln\left(\frac{\tau_{m}}{\tau_{0}}\right)} \end{array}$$

The blocking temperature does not only depend on the measurement time $\tau_{m}$, it also depends on the applied magnetic field *H* [32,33,34]:

(7) $$\begin{array}{c} T_{B}=\frac{KV}{k_{B}\ln\left(\frac{\tau_{m}}{\tau_{0}}\right)}{\left(1-\frac{H}{H_{K}}\right)}^{\frac{3}{2}} \end{array}$$

where $H_{K}$ is the is the anisotropy field, also called the switching field, which is defined as [32,33,34,35]:

(8) $$\begin{array}{c} H_{K}=\frac{2K}{M_{S}} \end{array}$$

Equation (7) is only valid for a non-interacting system, where each particle is separated such that the dipolar interactions can be neglected due to the spatial distance between each particle. In the framework of the random anisotropy model (RAM), the field-dependent correlation length, $L_{\mathrm{corr}}$, defines a volume where the effective anisotropy constant, *K*, results from averaging over all particles within such a volume [33,34]:

(9) $$\begin{array}{c} L_{\mathrm{corr}}=D+{\left(\frac{2A}{M_{S}H+C}\right)}^{\frac{3}{2}} \end{array}$$

where *A* is the intergranular exchange constant for nanocrystalline alloys and represents the effective interaction intensity [36]. The parameter *C* takes the influence of the particle concentration into account. For clustered particles, *C* is close to zero and increases with increasing separation of the particles, reaching

(10) $$\begin{array}{c} C\approx 2A-M_{S}H \end{array}$$

for a non-interacting particle system [33,37]. The parameter *C* was introduced to overcome the divergence for H=0 [34]. Using the RAM, Equations (7) and (8) can be rewritten for coupled particles as [33,34]:

(11) $$\begin{array}{c} T_{B}=\frac{K\pi \left[D^{3}+x\left(L_{\mathrm{corr}}^{3}-D^{3}\right)\right]}{6k_{B}\ln\left(\frac{\tau_{m}}{\tau_{0}}\right){\left[1+\frac{x}{D^{3}}\left(L_{\mathrm{corr}}^{3}-D^{3}\right)\right]}^{\frac{1}{2}}}\cdot {\left[1-\left(\frac{HM_{S}{\left[1+\frac{x}{D^{3}}\left(L_{\mathrm{corr}}^{3}-D^{3}\right)\right]}^{\frac{1}{2}}}{2K}\right)\right]}^{\frac{3}{2}} \end{array}$$

where *x* is the volume fraction that the particles occupy in the ensemble [34]. One can see that for high magnetic fields, *H*, or under the approximation of $C\approx 2A-M_{S}H$, the equation for the interacting particles (Equation (11)) is simplified to the non-interaction equation (Equation (7)). The reported values for anisotropy constants for nanoparticle systems based on iron oxides are usually in the range of 10 to 100 kJ/m${}^{3}$ [38].

The size dependence of the blocking temperature is clear from the above equations. As any real system has a size distribution of particles; consequently, a blocking temperature distribution has to be considered. Micha et al. showed a way to obtain an estimation of the blocking temperature distribution from zero-field cooling (ZFC) and field cooling (FC) measurements by calculating the first derivative of the difference of the ZFC and FC curve [39]:

(12) $$\begin{array}{c} f\left(T\right)\propto \frac{d\left(M_{\mathrm{ZFC}}\left(T\right)-M_{\mathrm{FC}}\left(T\right)\right)}{dT} \end{array}$$

This approach was used and confirmed by several research groups [31,34,40]. As any system of nanoparticles consists of a size distribution, it seems reasonable to identify the term "blocking temperature" as the temperature where the majority of the particles are in the blocked state, thus where the integral of Equation (12) reaches half of its maximum value [34].

## 3. Results

**Figure 1:**
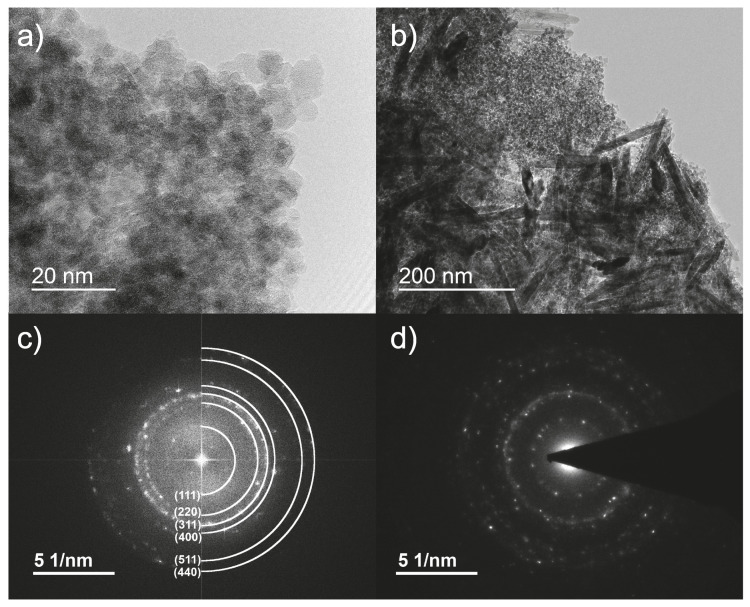
(**a**) High-resolution transmission electron microscopy image of iron oxide nanoparticles, (**b**) High-resolution transmission electron microscopy image of goethite nanowires, (**c**) FFT pattern of magnetite/maghemite nanoparticles calculated from (**a**,**d**) Selective area electron diffraction pattern of goethite nanowires.

**Figure 2:**
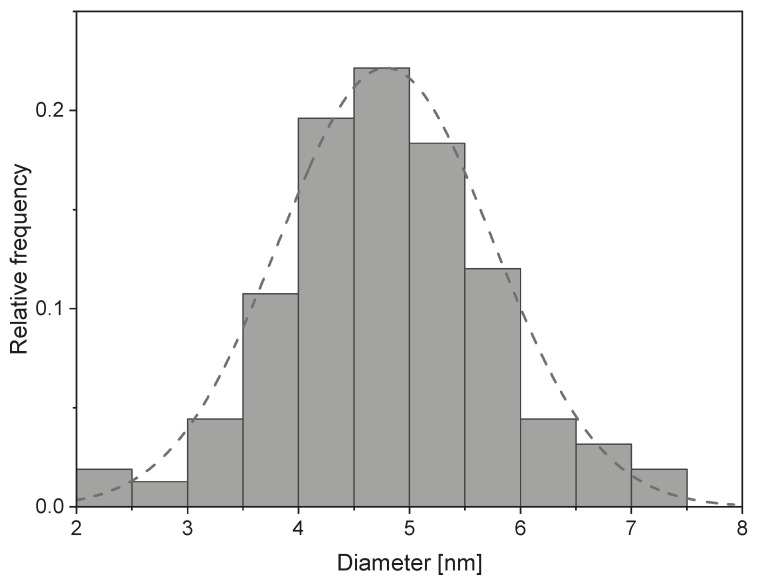
Nanoparticle size distribution for all doses. The dashed line shows a normal distribution fit (〈d〉=4.8 nm, σ=1.0 nm).

**Figure 3:**
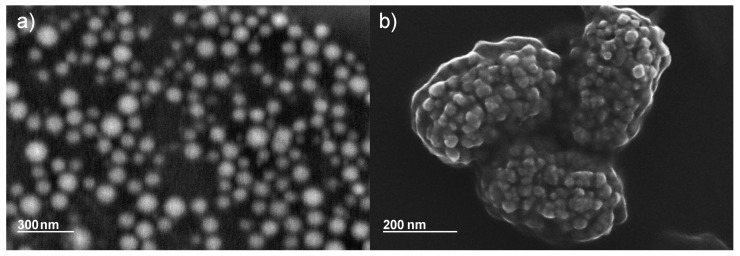
(**a**) Scanning electron microscopy image (4 keV) of dispersed nanoclusters after 250 kGy electron beam irradiation (**b**) Scanning electron microscopy image (15 keV) of agglomeration of nanoclusters after 150 kGy electron beam irradiation.

After irradiation, the suspension was a dark red-brownish colour. Figure 1a,b shows TEM images of the obtained spherical nanoparticles and a nanowire structure, which will be discussed later. Additional images for all irradiation doses can be found in the Supplementary Material (Figure S1). A visible nanoparticle formation could already be observed starting at doses of 50 kGy, but with a high portion of amorphous particles and a low yield. The synthesized particles have a spherical shape and an average size of 4.8 nm ± 0.9 nm (Figure 2). No significant difference in particle size for different doses could be observed. However, by increasing the irradiation dose (starting at 150 kGy), the particles tended to form clusters of increasing size. Figure 3 shows SEM images of samples after irradiation, taken from the solution without centrifugation and cleaning. Residues of the solvent caused charging effects (bright, foggy areas in the images). This made it necessary to reduce the acceleration voltage of the SEM, resulting in a reduced resolution.

**Figure 4:**
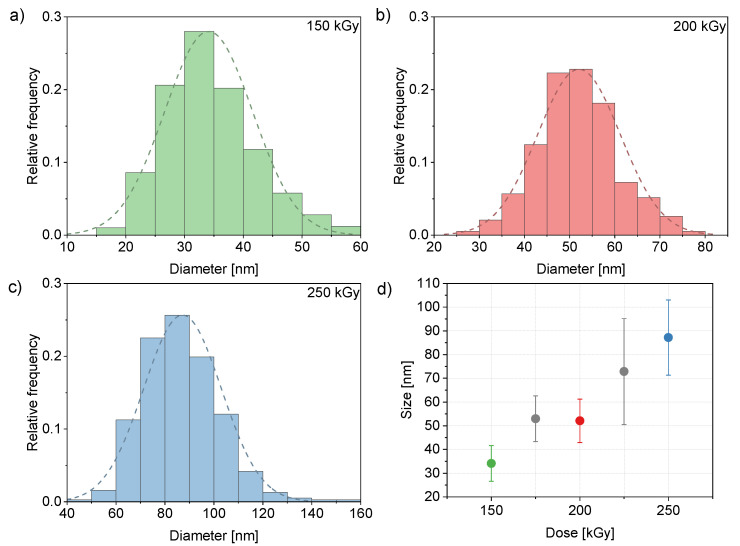
Cluster size distribution with normal distribution fit (dashed line) at (**a**) 150 kGy, (**b**) 200 kGy and (**c**) 250 kGy and (**d**) cluster size depending on irradiation dose.

Figure 3b shows an agglomeration of several clusters of 30 nm to 40 nm. Figure 4a–c shows the increasing size of the clusters with the increasing dose. For sufficient statistics, several separated areas of nanoclusters with more than 1100 particles in total were analysed. The clusters were approximated with an elliptical shape and the average of the two axes of the ellipse was taken as the particle diameter. The graph in Figure 4d shows the linear dependency of the cluster diameter, *D*, with the irradiation dose, Φ, yielding an approximate rate of dD/dΦ≈ 0.47 nm/kGy.

**Figure 5:**
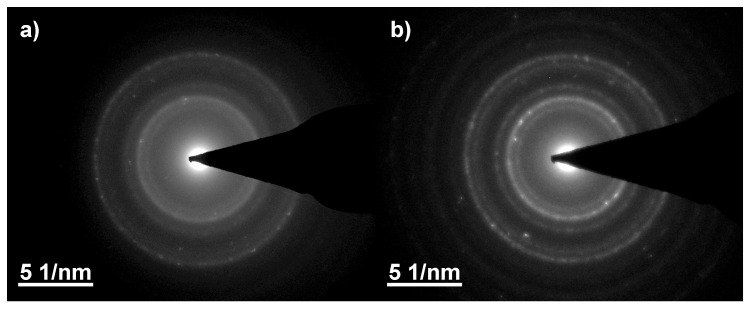
The selective area electron diffraction patterns for (**a**) 50 kGy and (**b**) 200 kGy irradiation dose show an increasing sharpness of the rings for increasing irradiation doses, indicating a higher crystallinity of the nanoparticles.

The crystalline phases were confirmed by selective area electron diffraction (SAED) and fast Fourier transformations (FFT) calculated from the corresponding high-resolution TEM (HRTEM) images. Figure 1c shows the FFT calculated from the HRTEM image presented in Figure 1a. The FFT image was indexed using inverse-spinel structures of magnetite and maghemite [41]. However, as the lattice distances of both iron oxides are very similar, distinguishing magnetite from maghemite is challenging. The calculated lattice constant of 8.419 Å ± 0.062 Å is closer to the one of magnetite (8.396 Å) than maghemite (8.345 Å) [9]. Electron irradiation favours crystallization of the particles; the diffractograms show a diffuse halo-ring pattern for low irradiation doses (Figure 5), which indicates a higher amount of disordered particles. With an increasing dose, the ring structure becomes clearer and sharper and shows a typical nanocrystalline pattern.

Besides nanoparticles, the formation of nanowires or rod-like structures with lengths of about 200 nm to 400 nm and widths of 10 nm to 25 nm could be observed (Figure 1b). Figure 1d shows the SAED pattern of such a structure and identifies it as goethite (FeO(OH)) with a characteristic d-spacing peak at 4.16 Å [41]. This goethite nanowires were visible in all samples regardless of irradiation dose. These structures were also reported by Gotić et al. after gamma radiation [15].

**Figure 6:**
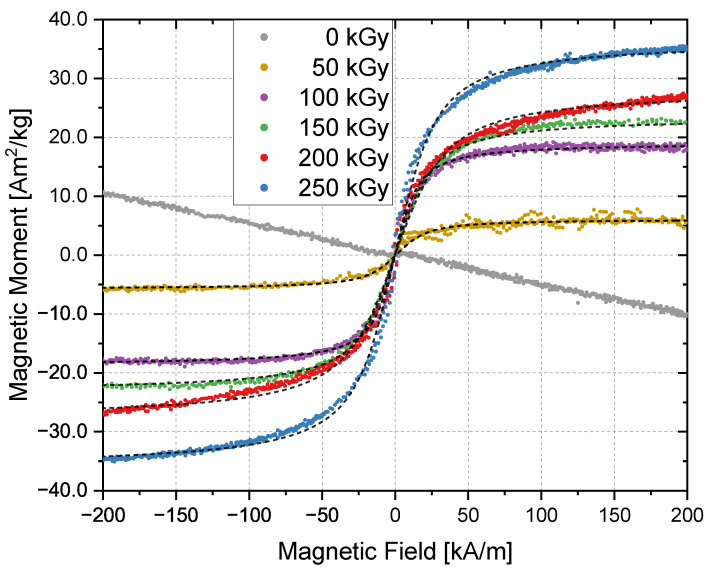
Magnetic moment depending on the applied magnetic field. The graphs from 50 kGy to 250 kGy are corrected for the diamagnetic part. The sample volume was 15 µL. The black dashed lines show a Langevin fit (Equation (4)) to each of the associated curves.

Magnetization measurements of the iron oxide nanoparticles in the presence of the solvent were performed using the VSM technique at 300 K and are shown in Figure 6. Complete cycles were performed in order to obtain potential hysteresis loops, but no coercivity or saturation remanence were detected. The unirradiated sample is characterized by diamagnetic behaviour, which is mainly caused by the solvent. This measurement was used to correct the diamagnetic contribution from all other magnetization curves (50 kGy to 250 kGy). The black dashed lines represent fits based on Equation (4) for each corresponding measurement. A saturation magnetization, $M_{\mathrm{SB}}$, of 80 Am${}^{2}$/kg was assumed as the mean value of the magnetite and maghemite values [9]. The obtained diameters from this approximation (Table 1) are in good agreement with the size distribution obtained by the TEM images, which corroborates the presence of superparamagnetic paricles. The samples irradiated with 50 kGy showed a high uncertainty. In some samples, no magnetic response could be detected or only signals with high noise were visible, as shown in Figure 6. With increasing irradiation doses, the saturation magnetization increases, which is an indicator for increasing crystallinity and a higher yield of magnetite/maghemite nanoparticles, since both have higher saturation magnetization than goethite (Fe_3_O_4_ ≈ 100 Am${}^{2}$/kg, γ−Fe_2_O_3_ ≈ 70 Am${}^{2}$/kg and FeO(OH) ≈ 1 Am${}^{2}$/kg) [9,11,42]. By normalizing the saturation magnetization with respect to the sample mass, one obtains 36 Am${}^{2}$/kg for the sample irradiated with 250 kGy, which is lower than the magnetization of pure magnetite or maghemite but still in a meaningful range. This could be an additional indicator that not all precursors are transformed to a magnetic iron oxide. The values are summarized in Table 1.

**Figure 7:**
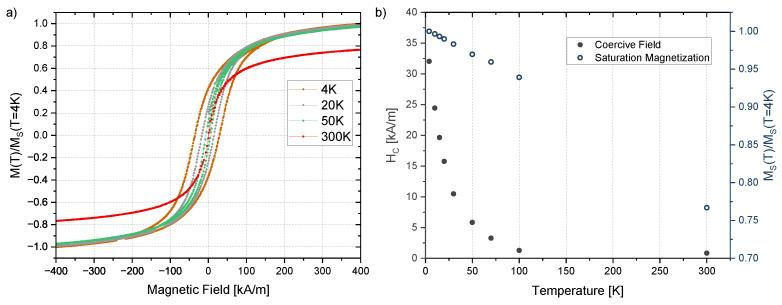
Temperature dependence of the magnetization for a sample irradiated with 200 kGy. (**a**) Magnetic moment depending on the applied magnetic field for temperatures down to 4 K and (**b**) coercive field (full points) and saturation magnetization (empty points) for temperatures between 4 K and 300 K.

For temperatures below 70 K, the magnetization curves start to show typical hysteresis loops (Figure 7a). The coercivity and the saturation magnetization increases with decreasing temperatures (Figure 7b).

**Figure 8:**
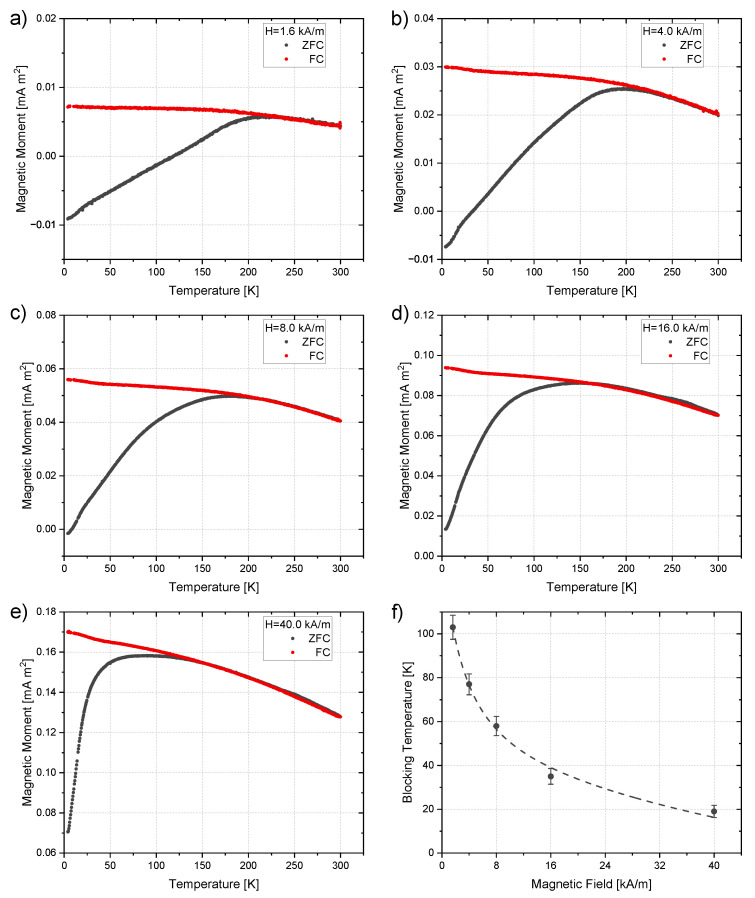
Zero-field cooling and field cooling curves of the 200 kGy sample for applied magnetic fields of (**a**) 1.6 kA/m, (**b**) 4 kA/m, (**c**) 8 kA/m, (**d**) 16 kA/m and (**e**) 40 kA/m. (**f**) Blocking temperature depending on the applied field. The dashed line shows a fit based on Equation (11) leading to an effective anisotropy constant of K=(21.2±1.7) kJ/m${}^{3}$.

In order to obtain the blocking temperature, $T_{B}$, and the effective anisotropy constant, *K*, zero-field cooling and field cooling measurements were performed. Figure 8 shows exemplary ZFC and FC curves for the sample irradiated with 200 kGy. One can clearly see the shift in the inflection point of the ZFC graph towards lower temperatures for higher applied fields. $T_{B}$ was determined from these curves as described in Section 2.4. By plotting these temperatures against the applied field (Figure 8f) and using the particle size obtained from TEM imaging (see Table 1), one can determine the efficient anisotropy constant, *K*, by fitting this data according to Equation (11). The values for all irradiation doses are summarized in Table 2 and are in a similar range to those reported by other studies [38,43,44]. The irradiation dose does not affect the magnetic anisotropy of the nanoparticles significantly. It is worth mentioning that for applied fields of 40 kA/m, the obtained values of *K* by using the equation for non-interacting particles (Equation (7)) show only small differences of around 2% compared to the one for interacting particles (Equation (11)). For lower fields (1.6 kA/m), the error bars increase above 50%, which underlines that Equation (7) can be a very good and straight-forward approximation for measurements performed at high magnetic fields.

**Figure 9:**
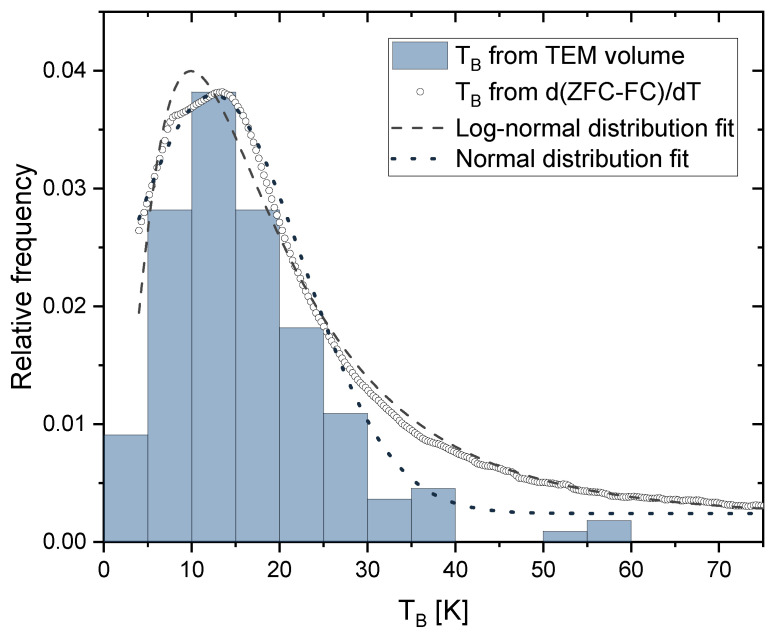
Blocking temperature distribution obtained by Equation (12) with log-normal fit ($\langle T_{B}\rangle =20.8$ K) and normal distribution fit ($\langle T_{B}\rangle =12.8$ K) together with calculated blocking temperature distribution based on Equation (7) obtained from the TEM size distribution and K=21.2 kJ/m${}^{3}$.

As mentioned in Section 2.4, an estimation of the blocking temperature distribution can be obtained by taking the derivative with respect to temperature of the difference between ZFC and FC curves (Equation (12)). Figure 9 shows an example of such a distribution for a sample irradiated with 200 kGy and an applied field of 40 kA/m. The resulting curves were fitted with a log-normal distribution function (dashed line in Figure 9), as suggested by Bruvera et al. [40]. Since the obtained size distribution (Figure 2) shows similarities with a normal distribution shape, an additional normal distribution curve was added for comparison (dotted line in Figure 9). The mean blocking temperature $\langle T_{B}\rangle$ was calculated from each distribution. The values for all irradiation doses are summarized in Table 2. By using the obtained value for *K*, the $T_{B}$ distribution can be merged with the size distribution obtained via TEM by using Equation 7, assuming a spherical shape of the nanoparticles. The histogram in Figure 9 shows the calculated blocking temperature based on the size distribution for the 200 kGy sample and is in very good agreement with the temperature distribution obtained via the approach of Micha et al. (Equation (12)), which is an additional confirmation of the determined anisotropy constant *K*.

## 4. Discussion

The radical reactions caused by irradiation processes are complex, and not all processes occurring in solution have been resolved at this point. Irradiation-induced hydrolysis of water molecules and simultaneous formation of secondary electrons, either from the water or the solvent, are the main contributions to particle formation as they influence the ratio of the Fe^2+^ and Fe^3+^ ions:

(13) $${\mathrm{Fe}}^{3+}+e_{\mathrm{aq}}^{-}\to {\mathrm{Fe}}^{2+}$$

This ratio has a high impact on the resulting products. A ratio smaller than 0.3 leads to enhanced goethite formation (Fe^3+^O(OH)) and ratios of 0.5 and higher are favourable in order to obtain magnetite particles [45,46]. There are two main reactions of formation of magnetite: the transformation from goehtite and the oxidation of Fe^2+^ and Fe^3+^ ions [47,48]:

(14) $$2{\mathrm{Fe}}^{3+}O\left(\mathrm{OH}\right)+{\mathrm{Fe}}^{2+}+2{\mathrm{OH}}^{-}\to {\mathrm{Fe}}^{2+}{\mathrm{Fe}}_{2}^{3+}O_{4}+2H_{2}O$$

(15) $$\;\;\;\;\;{\mathrm{Fe}}^{2+}+2{\mathrm{Fe}}^{3+}+8{\mathrm{OH}}^{-}\to {\mathrm{Fe}}^{2+}{\mathrm{Fe}}_{2}^{3+}O_{4}+4H_{2}O$$

The formation of maghemite would mainly result from the oxidation of magnetite [10,49]:

(16) $${\mathrm{Fe}}_{3}O_{4}+2H^{+}\to \gamma -{\mathrm{Fe}}_{2}O_{3}+{\mathrm{Fe}}^{2+}+H_{2}O$$

Acidic and anaerobic conditions are favourable for this transformation process as surface Fe^2+^ ions are desorbed as hexa-aqua complexes [10]. Additionally, temperatures higher than 200 °C enhance the transformation process of magnetite to maghemite [50]. The temperature during the process was much lower (50 °C to 60 °C) due to cooling by crushed ice during the irradiation, and the pH value was in the basic region. However, local oxidation at the outer shell of magnetite nanoparticles cannot be excluded and may result in formation of a core/shell structure [13]. In general, it is difficult to distinguish between magnetite and maghemite, because both have an inverse-spinel structure with similar lattice spacing [14,51]. A common way to describe the unit cell of magnetite is (Fe^3+^)_8_[Fe^2.5+^]_16_ O_32_, where the round brackets describe the tetrahedral sites and the square brackets describe the octahedral sites [52]. The Fe^2+^ ions are located at the octahedral sites and the electrons are constantly transferred between the Fe^2+^ and Fe^3+^ ions. By creating 8/3 vacancies out of the 24 Fe sites in the cubic unit cell of magnetite, here displayed as □, the maghemite structure ${{\left(\mathrm{Fe}\right)}_{3}}^{+}[{\mathrm{Fe}}_{5/6}^{3+}{□}_{1/6}{]}_{16}O_{32}$ is obtained [52,53]. One can see that the vacancies are located at octahedral sites and therefore the structure of maghemite can be approximated as a cubic unit cell, which results in similar SAED patterns, especially if nonstoichiometric magnetite (Fe_3−*x*_O_4_) or a mixture of magnetite and maghemite is present [52,53,54]. The obtained lattice constant for the iron oxide nanoparticles is closer to the lattice constant of magnetite; therefore, one could conclude that this iron oxide phase predominates. Still, it is quite likely that both crystalline phases are formed [49]. For further ferrogel applications, it is not necessary to obtain the exact magnetite/maghemite ratio, since the required properties (biocompatibility and magnetic behaviour) are very similar. Mössbauer spectroscopy could be a suitable tool to determine the oxidation state of iron species, but Santoyo Salazar et al. showed that there are limitations in accurately quantifying Fe^2+^ and Fe^3+^ if the size of the nanoparticles is smaller than 20 nm, because superparamagnetic relaxation effects cause line broadening and overlap, which results in an unresolved hyperfine structure [55]. Hah et al. performed Mössbauer spectroscopy with particles smaller than 10 nm and successfully identified the phase, but only for pure magnetite [56]. Frison et al. showed a way to distinguish between these two phases by synchrotron X-ray total scattering methods and an additional Debye function analysis of the magnetic data [57]. Kim et al. presented a procedure to obtain the ratio of the iron oxides by XRD with calibration curves based on predefined commercial available magnetite and maghemite powder samples [58], which unfortunately did not lead to clear results for our samples.

Both iron oxide phases are ferrimagnetic, but magnetite has a higher saturation magnetization than maghemite [9]. Goethite is an antiferromagnetic material for temperatures below 400 °C (Néel temperature) [59]. Since the VSM measurements were performed at room temperature, goethite’s contribution to the magnetic behaviour is small compared to the iron oxides. The increasing saturation magnetization with increasing dose (Figure 6) and the clearer diffraction patterns (Figure 5) lead to the interpretation that more magnetite/maghemite is formed and more goethite is transformed with the increasing dose. Furthermore, it indicates the growth of larger clusters, as the saturation magnetization increases with the overall structure size [60]. It is known that magnetite/maghemite particles with sizes smaller than approximately 10 nm show superparamagnetic behaviour [14,61,62,63]. The measured M(H) curves and approximations by the Langevin equation are consistent with that. As a result of sizes of around 5 nm of the smallest structures (Figure 1a), a single-domain magnetic character of the particle is expected. Due to thermal relaxation, no permanent magnetization occurs, and thus no hysteresis is visible in the graph, which one would expect for ferrimagnetic materials. Ge et al. [62,63] showed that clusters with sizes up to 180 nm show superparamagnetic behaviour, as each of these clusters consist of many single crystallites of approximately 10 nm in size. This observation can also be applied to our nanoclusters, as these also consist of smaller particles. The polyvinyl chains attached to the nanoparticles keep them apart and reduce dipolar interactions [13], which may result in a weaker bonding of the clusters on the one hand and, on the other hand, promote the relaxation of the magnetic moments inside the large clusters.

The anisotropy constant *K* is influenced by the particle size and shape. The size of the particles does not differ significantly for different irradiation doses and therefore it is reasonable that there is no change in the magnetic anisotropy. The determination of the anisotropy constant via Equation (7) or Equation (11) implies some approximations. The saturation magnetization, $M_{S}$, is considered to be constant inside the region of interest. This assumption is reasonable as the change in the saturation magnetization in the range of the determined blocking temperatures (4 K to 100 K, Figure 8f) is only about 7% (Figure 7b). Another approximation was performed by using the average diameter obtained by TEM imaging as the input parameter for the fitting function. Since the size of most of the obtained nanoparticles is close to the average diameter and, moreover, there is a good agreement between size and blocking temperature distribution (Figure 9), this approximation also seems valid. There is still some uncertainty depending on the used approach to obtain the mean blocking temperature (normal or log-normal distribution, 50% criteria). Both the normal and log-normal fitting show good agreement in the peak area of the $T_{B}$ distributions, with the log-normal curve showing a better agreement in the flattened regions of the distribution (Figure 9). The choice of criteria for defining $T_{B}$ affects the determination of *K* and since the mean values obtained from the distributions have some variation, we used the argument of half of the integral maximum to determine $T_{B}$ (Section 2.4). Besides the presented way of using a blocking temperature distribution, other criteria such as the inflection point or the maximum of the ZFC curves are used [40]. These criteria would lead to a much higher $T_{B}$ and consequently to higher *K* values. However, it should be noted that the effective anisotropy constant itself is also temperature dependent [64].

Compared to other publications which use gamma irradiation, a size difference of the nanoparticles with increasing doses could not be observed [15,17,48,65]. According to Belloni et al. [19], the size is mainly influenced by the irradiation rate and by certain polymers with functional groups that attach to the nanoparticles. In electron beam-assisted synthesis, the rate is very high and differs only marginally for the different doses. Additionally, a high irradiation rate may have a destabilizing effect on polyvinyl chains [66]. These are part of the surfactant (Triton X-100) that usually restricts the size of the particles. By partial destabilization of these chains, the growth of nanoparticles is not hindered, and the growth of large clusters, which is visible in the SEM images, is promoted. One also has to consider that the whole irradiation process with electrons takes just a few minutes instead of several hours of gamma irradiation. It is not clear how stable the cluster formations are and how the polyvinyl chains affect the stability, and therefore it cannot be excluded that the clusters were destroyed due to the cleaning procedure, which includes several steps of centrifugation and ultrasound baths.

The magnetic response started weakly at 50 kGy and was difficult to detect. Not all samples, which were irradiated with this dose, showed a magnetic response in our measurement system, most likely due to the detection limits of the VSM system (approx. $10^{-9}$ Am${}^{2}$). Referring to the SEM and TEM images, the amount of nanoparticles was small. Thus, the obtained $T_{B}$ and *K* values for the 50 kGy samples should be treated with caution. The yield and therefore the magnetic response increase drastically with irradiation doses of 100 kGy or higher, which is in agreement with Abedini et al., who reported an enhanced particle formation starting at 100 kGy for gamma irradiation [48]. This leads to the assumption that certain conditions have to be fulfilled in order to obtain magnetite either by transition from goethite (Equation (14)) or directly from the reaction of Fe^2+^ and Fe^3+^ with OH^−^ (Equation (15)). These conditions can be based on stoichiometry and an increased probability of ion collisions. The reducing environment caused by irradiation favours formation of magnetite instead of maghemite [15] and also enhances the electron transfer between Fe^2+^ and Fe^3+^, which plays a fundamental role in the formation of iron oxides with spinel structures [47,67]. Delocalization of electrons rearranges local structures, promotes spinel ordering and also supports dissolution–recrystallization processes [67]. A high molar ratio of the Fe^2+^/Fe^3+^ ions (greater than 0.5) in the reactants enhances these processes [45], which then leads to high magnetite nanoparticle formation and therefore a high probability to form clusters.

## 5. Conclusions and Outlook

Superparamagnetic iron oxide nanoparticles with a size of 5 nm were successfully synthesized by irradiating iron-containing micro-emulsions with an electron beam. An increasing irradiation dose increased the cluster size and the saturation magnetization, which indicates a higher yield of nanoparticles with increasing dose. The distributions of blocking temperatures were obtained from the ZFC and FC measurements, which led to the determination of the anisotropy constants for each sample. The magnetic anisotropy constant, similar to the particle size, was not affected by different irradiation doses and was in the range of K≈20 kJ/m${}^{3}$. SAED patterns identified the material as magnetite/maghemite, with clearer patterns for higher doses. Even though the lattice constant was closer to magnetite, which could imply that this iron oxide is predominant, it cannot be clearly assigned to one phase or the other. Most likely both oxides are present, which would still not affect the desired applications due to the similar properties of both iron oxides. Additionally, goethite nanowires could be observed regardless of the irradiation dose. In accordance with the increasing saturation magnetization and the increasing crystallinity revealed by the SAED patterns, we assume that the there are still some amorphous iron oxides in the sample and the amount is reduced by increasing the irradiation dose. Energetic electron (10 MeV) irradiation is demonstrated to be a powerful tool for the synthesis of magnetic nanoparticles and to modify the material properties. Electron irradiation is not only faster than gamma irradiation but also opens up possibilities to functionalize the particles or create composite materials for further applications. For example, Seino et al. immobilized small gold particles on bigger iron oxide nanoparticles by electron irradiation [68], which makes them interesting as magnetic particles with inert docking points for bio-molecules. As the cross-linking of magnetic particles with gels is performed by electron irradiation, one could combine these two processes to create new composite materials with unique properties.

## Figures and Tables

**Table 1:** Cluster size, saturation magnetization and particle diameter obtained from fitting and from TEM images for different doses.

Irradiation Dose (kGy)	Cluster Size (nm)	Saturation Magnetization (Am${}^{2}$/kg)	Particle Size from Equation (4) (nm)	Particle Size from TEM (nm)
50	-	6	5.4 ± 0.3	4.8 ± 0.5
100	-	18	5.9 ± 0.4	5.4 ± 0.7
150	37 ± 7	23	5.1 ± 0.3	4.7 ± 0.8
200	52 ± 9	28	4.6 ± 0.3	5.0 ± 0.6
250	87 ± 16	36	4.9 ± 0.4	4.9 ± 0.8

**Table 2:** The blocking temperatures, $T_{B}$, for an applied field of 40 kA/m were determined by a log-normal distribution fit, a normal distribution fit and by taking 50% of the integral maximum, as described in Section 2.4. The latter was used to obtain the effective anisotropy constant *K*.

Irradiation Dose (kGy)	$\langle T_{B}\rangle$ by Log-Normal Distribution (K)	$\langle T_{B}\rangle$ by Normal Distribution (K)	$T_{B}$ by 50% Maximum of Integral (K)	*K* (kJ/m^3^)
50	10.3 ± 0.3	8.9 ± 0.2	9.9 ± 0.3	17.9 ± 0.2
100	17.7 ± 0.4	11.5 ± 0.2	14.4 ± 0.3	18.1 ± 0.2
150	22.8 ± 1.8	11.6 ± 0.4	16.7 ± 0.4	22.4 ± 0.7
200	20.8 ± 0.9	12.8 ± 0.3	17.6 ± 0.4	21.2 ± 1.7
250	18.3 ± 2.8	8.1 ± 0.9	14.3 ± 0.3	20.0 ± 2.1

## Data Availability

The data that support the findings of this study are available from the corresponding authors upon reasonable request.

## Supplementary Materials

The following supporting information can be downloaded at: https://www.mdpi.com/article/10.3390/nano13050786/s1, Figure S1: HRTEM images of iron oxide nanoparticles and nanowires for irradiation doses from 50 kGy to 250 kGy.

## Abbreviations

The following abbreviations are used in this manuscript:

SEM	Scanning electron microscopy
VSM	Vibrating sample magnetometer
TEM	Transmission electron microscopy
HRTEM	High-resolution transmission electron microscopy
SAED	Selective area electron diffraction
FFT	Fast Fourier transformations
RAM	Random anisotropy model
ZFC	Zero-field cooling
FC	Field cooling
