# Supplementary material for: Energetic Electron-Assisted Synthesis of Tailored Magnetite (Fe3O4) and Maghemite (γ−Fe2O3) Nanoparticles: Structure and Magnetic Properties
Source: Nanomaterials (Basel). 2023 Feb 21;13(5):786. doi: 10.3390/nano13050786 (PMC10005483; doi:10.3390/nano13050786)
Supplement: Supplementary file 1 [file nanomaterials-13-00786-s001.zip › nanomaterials-2213146-supplementary.pdf]

Supplementary Material

Energetic Electron-assisted Synthesis of Tailored Magnetite (Fe_3O_4) and Maghemite (-Fe_2O_3) Nanoparticles: Structure and Magnetic Properties

Johannes Dietrich [1,2,*] , Alexius Enke [1,2], NilsWilharm [1,2] , Robert Konieczny [1], Andriy Lotnyk [1] , André Anders [1,3] and Stefan G. Mayr [1,2,*]

1 Leibniz Institute of Surface Engineering (IOM), Permoserstraße 15, 04318 Leipzig, Germany
2 Division of Surface Physics, Faculty of Physics and Earth Science, Leipzig University, Linnéstraße 5, 04103 Leipzig, Germany
3 Division of Applied Physics, Faculty of Physics and Earth Science, Leipzig University, Linnéstraße 5, 04103 Leipzig, Germany
***** Correspondence: johannes.dietrich@iom-leipzig.de (J.D.); stefan.mayr@iom-leipzig.de (S.G.M.)

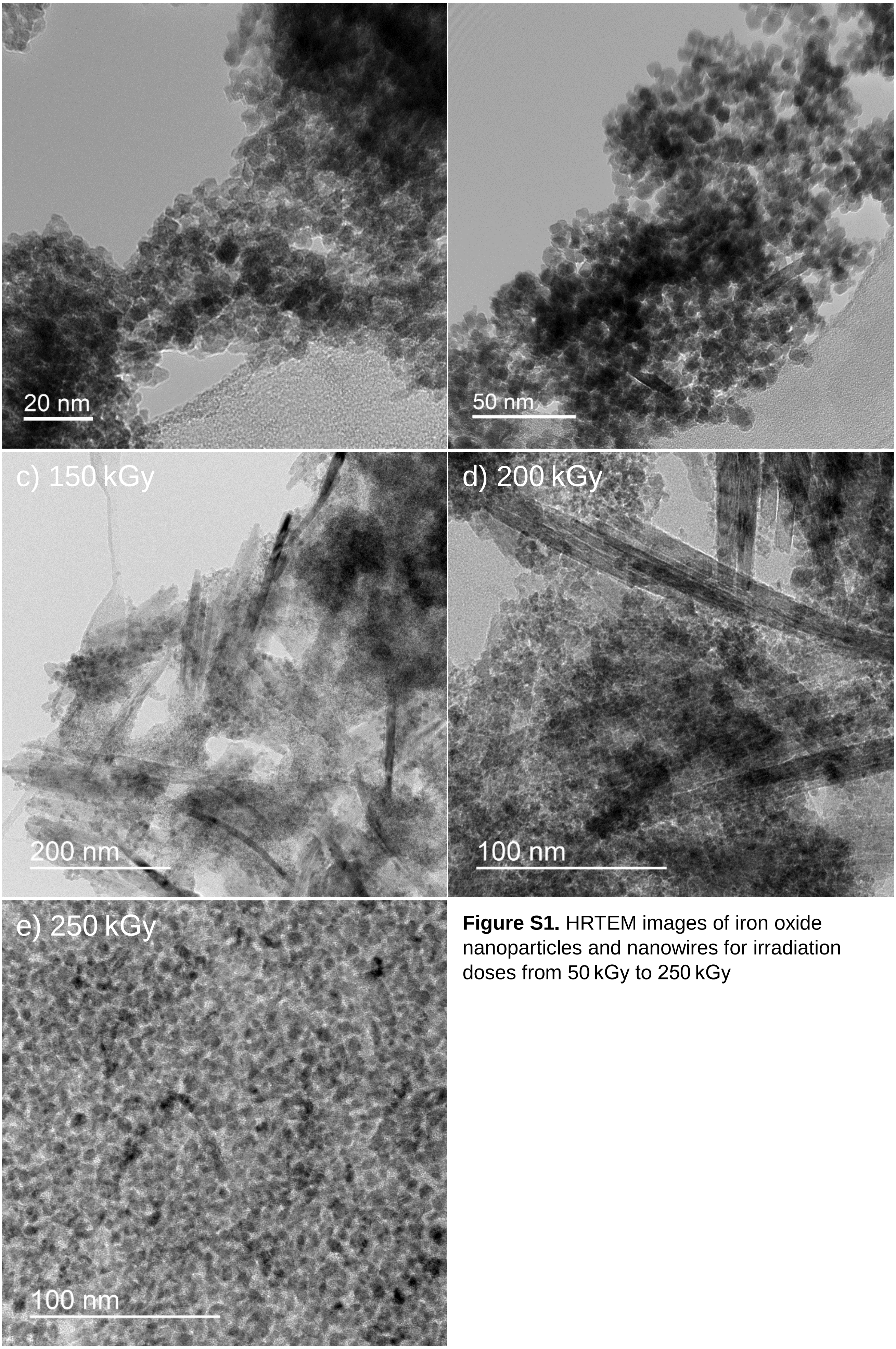

Figure S1. HRTEM images of iron oxide nanoparticles and nanowires for irradiation doses from 50 kGy to 250 kGy
